# The first fungi: mode of delivery determines early life fungal colonization in the intestine of preterm infants

**DOI:** 10.20517/mrr.2021.03

**Published:** 2022-01-28

**Authors:** Jannie G. E. Henderickx, Heleen de Weerd, Liesbeth J. Groot Jebbink, Diny van Zoeren-Grobben, Marieke A. C. Hemels, Richard A. van Lingen, Jan Knol, Clara Belzer

**Affiliations:** ^1^Laboratory of Microbiology, Wageningen University and Research, Stippeneng 4, Wageningen 6708 WE, The Netherlands.; ^2^Danone Nutricia Research, Uppsalalaan 12, Utrecht 3584 CT, The Netherlands.; ^3^Isala Women and Children’s Hospital, Department of Neonatology, Dokter van Heesweg 2, Zwolle 8025 AB, The Netherlands.

**Keywords:** Intestinal tract, gut, fungi, mycobiota, premature birth, infant

## Abstract

**Aim:** The role of intestinal fungi in human health and disease is becoming more evident. The mycobiota composition and diversity of preterm infants is affected by interactions with bacteria and clinical variables. In this study, we aimed to characterize the composition and the diversity of the preterm infant mycobiota and the effect of clinical variables on it in the first six postnatal weeks.

**Methods:** Preterm infants (*n *= 50) and full-term infants (*n* = 6) admitted to Isala Women and Children’s hospital (Zwolle, The Netherlands) who were born during 24-36 or 37-40 weeks of gestation, respectively, were included in this study. Feces were collected during the first six postnatal weeks (*n* = 109) and their mycobiota composition and diversity were characterized by ITS2 amplicon sequencing.

**Results:** Composition analyses identified fungi and other eukaryotic kingdoms, of which Viridiplantae was most abundant. Of the fungal kingdom, Ascomycota and Basidiomycota were the first and second most prominent phyla in early life of all infants. *Candida* was the most abundant genus in the first six weeks of life and increased with gestational and postnatal age. Fungal phylogenetic diversity remained stable in the first six postnatal weeks. The individuality and the mode of delivery were identified as significant predictors for the variation in the mycobiota composition. Vaginally delivered infants were enriched in *Candida* spp., whereas infants delivered through emergency C-section were characterized by *Malassezia* spp.

**Conclusion:** These results indicate that fungi and other eukaryotic kingdoms are detected in the intestine of preterm and full-term infants in the first six postnatal weeks. Similar to the microbiota, colonization of the preterm intestine with fungi is determined by clinical variables including individuality and mode of delivery.

## INTRODUCTION

The human gastrointestinal tract harbors bacteria, fungi, archaea, protozoa, and viruses that together form the microbiota^[1]^. Most research has emphasized the relationship between the bacterial part of the microbiota and its link to health or disease^[2-4]^. By comparison, little is known about the fungal part of the microbiota, which collectively is called the “mycobiota”. The necessity to investigate the microbiota beyond bacteria is becoming more evident, as “interkingdom” interactions in the intestine can affect ecosystem dynamics and immune homeostasis^[1,5]^.

The initial fungal colonization occurs during early life and the process is very similar to that of the microbiota. The acquisition of the first fungi may occur by vertical transmission from mother to infant, in which *Candida* spp. is most extensively studied in this regard^[6,7]^. After birth, the mycobiota composition and diversity is affected by variables very similar to those affecting the microbiota. They include gestational age, mode of delivery, hospital environment, antibiotic exposure, and diet^[8-11]^.

The mycobiota composition and diversity of preterm infants may be considerably different compared to full-term infants due to aberrant circumstances in early life. Apart from their direct impact, those aberrant circumstances may affect the mycobiota indirectly through interkingdom interactions^[1,12,13]^. The preterm infant gut mycobiota, in contrast to healthy full-term infants, is often dominated by a single species^[11]^. Yeasts, and more specifically *Candida* spp., are typically one of those predominant species in preterm infants up to a postnatal age of six months^[11,14]^. Within the *Candida* genus, opportunistic pathogens *Candida albicans* and *Candida parapsilosis* are highly prevalent and persistent in preterm infants^[11]^. Other dominant genera identified in preterm infants include *Aspergillus*, *Davidiella*, *Debaryomyces Penicillium,* and *Saccharomyces*^[11]^. In addition, fungi of the *Saccharomycetales* order and species of the *Cladosporium* and *Cryptococcus* genus have been identified in stools of extremely low birth weight and preterm infants^[14,15]^.

While many intestinal fungi are commensal and may confer health benefits, fungal overgrowth may lead to infections that are associated with considerable morbidity and mortality rates^[9,16,17]^. Preterm infants are particularly prone to invasive, systemic candidiasis that affects approximately 10% of preterm infants and has an associated mortality rate of 20%^[18,19]^. The susceptibility to fungal overgrowth in preterm infants correlates to predisposing clinical factors including a naïve immune system, bacterial dysbiosis following exposure to a hospital environment, antibiotic treatment, and use of parenteral nutrition^[9]^.

In this study, we aimed to characterize the composition and diversity of the preterm infant mycobiota and the effect of clinical variables on it during the first six postnatal weeks. We investigated the fecal mycobiota of infants born with varying degrees of prematurity during the first six postnatal weeks.

## METHODS

### Ethics declaration

The board of the Medical Ethics Committee (METC) of Isala Zwolle concluded that this study does not fall under the scope of the Medical Research Involving Human Subjects Act (WMO). Informed consent was obtained from both parents of all individual participants included in the study.

### Study description

The samples from this study derive from the EIBER study; a single-center, observational study involving full-term and preterm infants admitted to the neonatal intensive care unit (NICU) or the pediatric ward of Isala Women and Children’s hospital in Zwolle, The Netherlands. The two objectives of the EIBER study were to investigate colonization and development of the gut microbiota and to understand the relationship between microbiota composition and antibiotic treatment duration^[20-23]^.

The preterm infants were fed with mother’s own milk when available, which was increasingly supplemented with human milk fortifier (Nenatal BMF, Nutricia, The Netherlands) starting at an intake of 100 mL/kg/day according to standard practice in Dutch NICUs. Whenever human milk was insufficient or not available, preterm infants were (mixed) fed with preterm formula (Nutrilon Nenatal Start, Nutricia, The Netherlands). Data on the percentage of human milk and formula feeding are available [Supplementary Table 1]. No donor milk bank was available at the NICU during the study period.

As part of the EIBER study, fecal samples of preterm and full-term were collected immediately after birth and during postnatal weeks 1, 2, 3, 4, and 6. Previously, these samples have been used to assess the composition and functionality of the preterm microbiome by means of 16S rRNA gene amplicon sequencing^[22,23]^ and metaproteomics^[20,21]^.

### Sample selection

Fecal samples were selected based on the following criteria:• Gestational age was between 24 and 40 weeks.• Mothers did not receive antibiotic treatment during labor until six weeks thereafter.• Infants received at least one antibiotic treatment.

The selection criteria were formulated to yield an as homogeneous as possible group. Infants were excluded if the mother received antibiotic treatment in the period of 48 h before birth until six weeks after birth. After infant selection, samples of some infants were unavailable or insufficient in volume at specific timepoints to conduct DNA extraction [Supplementary Table 2]. This resulted in a total of 116 fecal samples from 57 infants for DNA extraction [Supplementary Figure 1].

### DNA extraction

DNA extraction was performed on feces. First, 0.13 g of feces were weighed into a 2.0 mL screw cap tube filled with 0.25 g of 0.1 mm zirconia beads and three 2.5 mm glass beads. The weighed samples were stored at -80 °C until further processing. Every run included randomly selected fecal samples as well as a negative control consisting of one empty FastPrep tube with beads. Then, 300 μL of Stool Transport and Recovery Buffer (S.T.A.R. buffer, Cat. No. 03335208001, Roche Diagnostics) were added and bead-beaten three times at 5.5 ms for 60 s with 15 s pause (FastPrep-24 5G bead beating grinder and lysis system, MP Biomedicals). Subsequently, samples were incubated for 15 min at 95 °C at 100 rpm after which they were centrifuged (4 °C, 5 min 14,860 rpm) and supernatant was stored at 4 °C. The process was then repeated with 200 μL S.T.A.R. buffer. In the case the first step did not yield supernatant, 300 μL of S.T.A.R. buffer were added. Subsequently, 250 μL of recovered supernatant were used for DNA extraction with Maxwell 16 Tissue LEV Total RNA Purification Kit (Cat. No. AS 1220, Promega). Isolated DNA was checked for quality with Nanodrop and quantified with Qubit dsDNA BR Assay Kit (Cat. No. Q32850, ThermoFisher Scientific) on DeNovix (DS-11 FX, DeNovix).

### Mock community

The Mycobiome Genomic DNA Mix (MSA-1010, ATCC) was used as mock community and was included in each sequencing library. The DNA-based mock community samples were derived from the same stock and were used as technical replicates. Species in the Mycobiome Genomic DNA Mix included *Aspergillus fumigatus* (ATCC MYA-4609D-5), *Cryptococcus neoformans* var. *grubli* (ATCC 208821D-2), *Trichophyton interdigitale* (ATCC 9533D-5), *Penicillium chrysogenum* (ATCC 10106D-5), *Fusarium keratoplasticum *(ATCC 36031D-5), *Candida albicans* (ATCC 10231D-5), *Candida glabrata* (ATCC 2001D-5), *Malassezia globose* (ATCC MYA-4612D-5), *Saccharomyces cerevisiae* (ATCC 201390D-5), and *Cutaneotrichosporon dermatis* (ATCC 204094D-5).

### Amplification and sequencing

Fecal samples, mock communities, and negative controls were sent to Novogene (Cambridge, United Kingdom). Isolated DNA was measured for DNA purity and concentration with Nanodrop and Qubit 2.0, respectively, and integrity was visually inspected by agarose gel electrophoresis. Subsequently, samples were PCR-amplified with primers targeting the Internal Transcribed Spacer (ITS) 2 region (ITS3-2024F GCATCGATGAAGAACGCAGC, ITS4-2409R TCCTCCGCTTATTGATATGC) according to Novogene’s protocol. Quality control of the PCR-amplified samples was performed by visual inspection of amplified PCR products after gel electrophoresis on agarose gel. Next, PCR products were mixed, purified, and randomly assigned to a library. Libraries were prepared with NEBNext Ultra IIDNA Library Prep Kit (Cat No. E7645, New England Biolabs). After quality control of the library, ITS amplicon metagenomic sequencing was performed on the Novaseq6000 platform with 250 paired-end reads and a sequencing depth of 30,000 raw tags/sample. The samples were sequenced in two independent sequencing runs, in which mock communities were included in each library as technical replicates. DNA Mocks 1-4 and 5-8 were present as technical replicates in the first and second libraries, respectively. Raw sequencing data were checked for distribution of sequencing quality and error rate. Raw sequences with barcode and primer removed and supporting metadata were deposited in the European Nucleotide Archive (http://www.ebi.ac.uk/ena) under the accession number PRJEB48004.

### Bioinformatics

#### Preparing a theoretical mock community

As quality control, a theoretical mock community was prepared and used to compare to the sequencing output of the Mycobiome Genomic DNA Mix. To this end, fasta sequences of the ITS region of fungal species in the Mycobiome Genomic DNA Mix were retrieved from the nucleotide database of NCBI. ITS sequences of each fungus were trimmed by aligning them with the primers in the MUSCLE alignment tool of MEGA X (version 10.1.8)^[24]^. A fake mock was then created with our in-house Python code (available at: https://gitlab.com/wurssb/gen_fake_mocks) by importing trimmed sequences as well as a file containing a barcode and a file containing proportions of species (10.0% each).

#### Taxonomic assignment with Qiime2

Raw reads were processed according to the Q2-ITSxpress workflow^[25]^. Raw reads without barcodes and primers were imported in Qiime2. Subsequently, the conserved regions around the ITS gene were trimmed with ITSxpress^[26]^, which has been shown to improve accuracy of taxonomic classification^[27]^. The sequence variants were then identified in the unmerged, trimmed sequences with Dada2^[28]^. Next, the Qiime classifier was trained using the UNITE database (version 8.3, all eukaryotes) with highest number of reference sequences (RefS) as compared to representative sequences (RepS)^[29]^. Fungal ITS classifiers were trained on the UNITE database on full reference sequences. Subsequently, sequence variants were classified with the trained classifier.

### Data analysis

#### Pre-processing

Data were imported in R version 3.6.3^[30]^ with the *Qiime2R* package (version 0.99.6)^[31]^ to make a phyloseq object. Before pre-processing, 10,596 taxa were identified in 129 samples with 9,216,861 reads in total. The average number of reads per sample were 71,449 with a minimum of 5 and a maximum of 138,138, showing high variability between samples. Pre-processing of the data included various steps, of which the first was filtering ASVs on kingdoms. Non-fungal kingdoms were removed and consisted of Alveolata, Chromista, Eukaryota kgd Incertae sedis, Metazoa, Stramenopila, and Viridiplantae [Supplementary Figure 2A and B]. However, unassigned sequences at kingdom level were retained. Next, as part of further downstream processing, 834 singletons (ASVs of which the sum of reads is equal to one) were removed. Subsequently, samples with reads below 1000 were omitted. Eight samples were omitted with reads below 1000 for further analyses [Supplementary Figure 1]. This resulted in a total of 121 samples, namely 109 fecal samples from 56 infants, 8 mocks, 1 theoretical mock, and 3 negative controls [Supplementary Figure 1, Supplementary Table 1]. Infants were categorized according to their gestational age into extremely preterm (24-27 weeks of gestation, *n* = 18 infants), very preterm (28-31 weeks of gestation,* n* = 15 infants), late preterm (32-36 weeks of gestation,* n* = 17 infants), and full-term (37-40 weeks of gestation, *n* = 6 infants). For each infant, the human milk intake was corrected for enteral feeding. To this end, the fraction of human milk intake was multiplied by the fraction of enteral feeding.

#### Composition plots

For the number of reads, the reads from the ASV table were used to generate composition plots. For the relative abundance composition plots, the data were first transformed to compositional data with the *transform* function from the *microbiome* package (version 1.8.0)^[32]^. Composition plots were visualized and customized with the *ggplot2* package (version 3.3.3)^[33]^.

#### Mock community check

Quality control was based on mock communities, in which compositions of DNA mocks were compared to each other and to the fake mock. First, normality of the number reads and relative abundance was checked visually with the *ggqqplot* function from the *ggpubr* package (version 0.3.0)^[34]^ and quantitatively with *shapiro.test* function from the *stats* package (version 3.6.3)^[30]^. The null hypothesis (normal distribution) was rejected in both cases as the *P*-values were smaller than 0.05.

First, compositions of DNA mocks were compared to each other based on the number of reads and the relative abundance. The number of reads between the deviating DNA Mock 1 and other DNA mock samples were compared with the *kruskal.test* from the *stats* package. Although the number of reads of DNA Mock 1 were lower, this did not yield significant difference compared to other DNA mock samples (*P* = 0.76, Supplementary Figure 3A). Next, technical replicates of the DNA mock communities were correlated with a Pearson correlation matrix using *pairs.panels* from the *psych* package (version 1.9.12)^[35]^. Correlation coefficients of the DNA Mock Technical Replicates 2-8 ranged between 0.85 and 1.00, indicating reproducibility of sequencing runs (*P *≤ 0.001, Supplementary Figure 4). As DNA Mock 1 was in the same library as the DNA Mocks 2-4, we deemed our data reliable.

Second, compositions of DNA mocks were compared to the fake mock. The same genera were identified in the DNA mock communities and the fake mock. However, some genera were over- or under-represented in the DNA mock communities. In the DNA mock communities, the mean relative abundances of *Fusarium*, *Candida*, and *Cutaneotrichosporon* were 0.22 ± 0.05, 0.16 ± 0.05, and 0.16 ± 0.07, respectively [Supplementary Figure 3B]. Compared to a theoretical relative abundance of 0.1 of each genus, they were the three most overrepresented genera in the DNA mock communities compared to the fake mock. On the other hand, compared to a theoretical relative abundance of 0.1 of each genus, *Trichopython*, *Aspergillus*, and *Malassezia* were the most underrepresented with relative abundances of 0.03 ± 0.01, 0.03 ± 0.01, and 0.01 ± 0.01, respectively [Supplementary Figure 3B]. Mean relative abundances of over- and under-represented genera in DNA mocks were not significantly different from the theoretical mock community (Mann-Whitney test, *P*-values not shown).

#### Hierarchical clustering

Data were rarified on the minimum sum of reads (1184) using the *rarefy_even_depth* function of the *microbiome* package. Distance was calculated with the *distance* function of the *phyloseq* package (version 1.30.0)^[36]^ using unweighted UniFrac and sample-wise comparisons. The *dendextend* package (version 1.13.4)^[37]^ was used for generating the hierarchical cluster plot.

#### Phylogenetic diversity

Phylogenetic diversity was calculated on rarified data with the *pd* function of the *picante* package (version 1.8.2)^[38]^. Significance was determined with the *compare*_*means *function of the *ggpubr *package with default settings except *P*-values were adjusted using BH correction. The plot was generated using the *ggplot2* package.

#### Redundancy analysis

Dimension reduction analysis was performed to identify clinical variables that significantly explained variation in the mycobiota composition. To this end, compositional data were transformed with centered log ratio (CLR) using the *transform* function of the *microbiome* package. Next, core members of the mycobiota were defined with *core_members* from the *microbiome* package with detection set to 1/1000 and prevalence set to 25/100. Detrended correspondence analysis was performed with *decorana* from the *vegan* package (version 2.5-6)^[39]^ to determine the correct dimension reduction method. Redundancy analysis (RDA) was performed with the *vegan* package, using Aitchison distance, defined as the Euclidean distance between CLR-transformed compositions^[40,41]^. CLR-transformed ASV relative abundances were not scaled. Samples with missing values of explanatory variables were omitted, leaving 95 samples as input. After running the first RDA, variance inflation was checked with *vif.cca* from the *vegan* package to omit clinical variables with VIF ≥ 10. Next, RDA was repeated, now with forward and reverse automatic stepwise model selection for constrained ordination with *ordistep* from the *vegan* package with settings p_in_ = 0.05, p_out_ = 0.1, and 999 permutations. Resulting *P*-values were adjusted with *p.adjust* from the *Stats* package using BH correction.

#### Permutational multivariate analysis of variance

Permutational multivariate analysis of variance (PERMANOVA) was performed with *adonis* from the *vegan* package to test for community-level differences between group centroids. CLR-transformed compositional data of the core mycobiota were used for this analysis. Permutations were set to 999 and Euclidean was used as dissimilarity matrix. Gestational age category was tested with PERMANOVA. Subsequently, homogeneity of variances was checked with *vegdist* and *betadisper* from the *vegan* package. For the gestational age categories, the outcome failed to reject the null hypothesis of homogeneous multivariate dispersions, and this predictor was therefore concluded to have homogenous multivariate dispersions.

#### Linear discriminant analysis effect size

Linear discriminant analysis effect size (LEfSe) was performed to assess differences between mode of delivery groups (vaginal delivery, planned C-section and emergency C-section) at phylum, class, order, family, genus, and species level. For this analysis, only fecal samples from preterm infants were selected (*n* = 96). The samples per mode of delivery groups were as follows: vaginal delivery *n* = 54; planned C-section *n *= 28; and emergency C-section, *n* = 14. The *phyloseq2lefse* function as provided on the *Rrumen* package GitHub^[42]^ was used on compositional data to generate the input file for Huttenhower lab Galaxy server (https://huttenhower.sph.harvard.edu/galaxy/root). The alpha value for the two-tailed non-parametric Kruskal-Wallis test was set to 0.01 and the logarithmic LDA score for discriminative features to 3.5. For multi-class analyses, the one-against-all method was selected.

## RESULTS

### Composition of fungal taxa in the preterm infant intestine over the first six postnatal weeks

**Figure 1:**
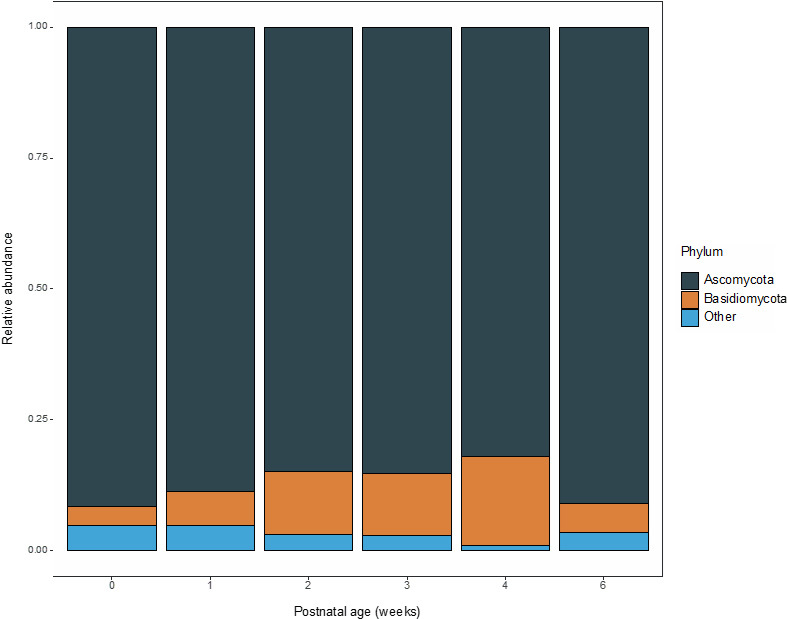
Relative abundance of the two most abundant fungal phyla in feces of preterm and full-term infants during the first six postnatal weeks.

Besides fungi, other eukaryotic kingdoms were observed and included Alveolata, Eukaryota kgd Incertae sedis, Chromista, Metazoa, Stramenopila, and Viridiplantae [Supplementary Figure 2A]. These kingdoms comprised 4283 taxa and 23.1% of total observed reads. For further analyses, the fungal and unassigned kingdoms were retained, after which the relative abundance of the fungal kingdom ranged between 95.3% and 99.2% during the first six postnatal weeks with the remainder being unassigned [Supplementary Figure 2B]. After pre-processing the data, the remaining fecal samples (*n* = 109) were further assessed for mycobiota composition. The first and second most abundant phyla in feces were Ascomycota and Basidiomycota, respectively [Figure 1]. Mean Ascomycota relative abundance varied between a minimum of 82.1% and a maximum of 91.7% (± 28.8% and ± 8.9% SD, respectively) in the first six postnatal weeks. Mean relative abundance of Basidiomycota gradually increased until the fourth week from 3.5% to 17.0% (± 4.2% and ± 28.6% SD, respectively), after which it decreased in the sixth week to 5.4% (± 11.5% SD). Both phyla were consistently the most dominant in preterm and full-term infants in all postnatal weeks. In twenty samples, Basidiomycota abundance was higher than the highest average of 17.0%. However, this could not be related to gestational or postnatal age.

### The relative abundance of *Candida* spp. increases with gestational and postnatal age

**Figure 2:**
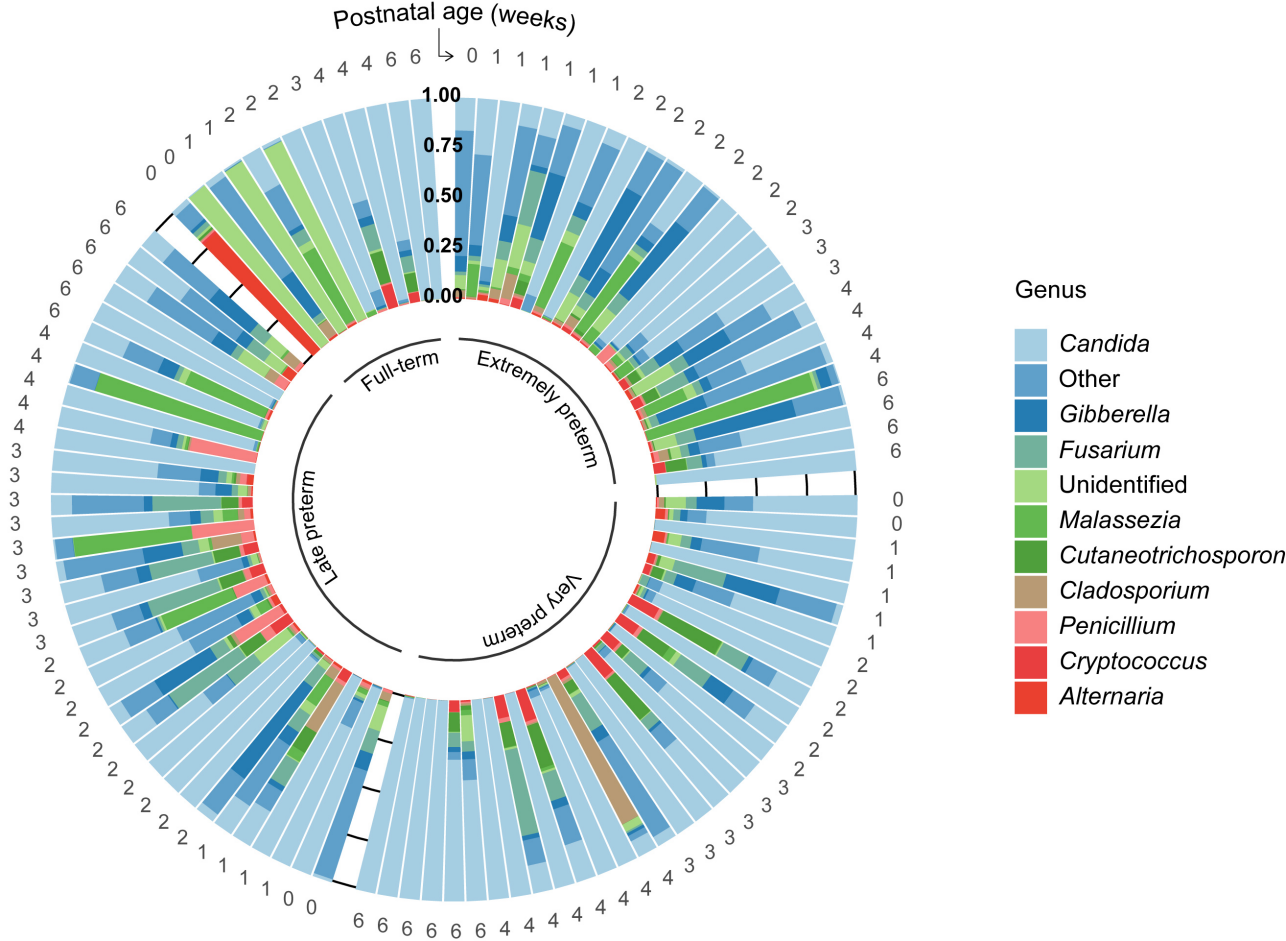
Relative abundance of the ten most abundant genera in every fecal sample of preterm and full-term infants. The postnatal age in weeks is displayed on the outer circle; the gestational age categories are displayed on the inner circle. The horizontal lines indicate the relative abundance in quartile percentages. Genera not belonging to the ten most abundant ones are merged under “Other”.

Within the Ascomycota phylum, *Candida* spp. was consistently the most abundant genus in the first six weeks. The genus was observed in all samples of preterm and full-term infants [Figure 2]. On average, it comprised approximately one third of observed genera in the first week (35.2% ± 40.0%) and up to more than two thirds in the last week (68.6% ± 36.3%), albeit with high variability between samples [Supplementary Figure 5A, Figure 2]. The total number of reads for this genus increased over time from 21,871.6 reads in meconium to 60,094.6 reads at Postnatal Week 6 [Supplementary Figure 6]. Of the *Candida* species, *C. albicans* was predominant with relative abundances ranging between 88.7% ± 21.5% (Week 1) and 96.5% ± 7.3% (Week 6) [Supplementary Figure 7].

Relative abundance of *Candida* spp. gradually increased both with gestational age category as well as postnatal age [Supplementary Figure 5B]. In extremely preterm infants, colonization with *Candida* spp. was most stochastic due to high standard deviations. The relative abundance of this genus increased from 0.39 in the first week to 0.56 in the sixth week in extremely preterm infants (± 0.38 and ± 0.41 SD, respectively), whereas *Candida* spp. increased from 0.02 in the first week to an abundance as high as 1.00 in full-term infants (± 0.02 and ± 0.00 SD, respectively, Supplementary Figure 5B).

### Phylogenetic diversity of the mycobiota remains stable in the first six postnatal weeks

**Figure 3:**
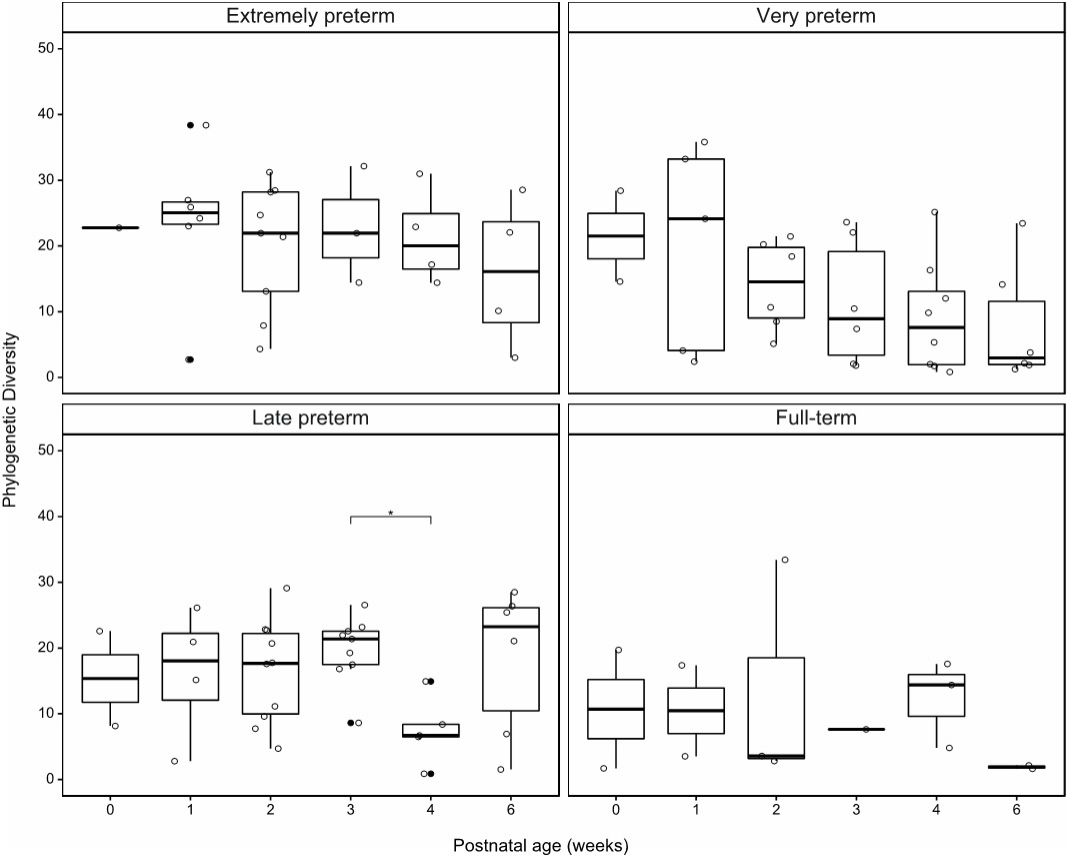
Phylogenetic diversity of preterm and full-term infants during the first six postnatal weeks. Individual data points are displayed as open circles, whereas outliers are filled circles. *P_adj_ ≤ 0.05.

Diversification of the mycobiota was investigated by performing phylogenetic diversity analyses in each gestational age group over the first six postnatal weeks [Figure 3]. Median phylogenetic diversity decreased from the first week onwards in extremely and very preterm infants, although none of these changes were statistically significant. Late preterm infants and full-term infants, who are physiologically most similar, were stable in phylogenetic diversity in the first two postnatal weeks. The number of samples in later postnatal weeks in the full-term infant group were too limited to be conclusive. Interestingly, phylogenetic diversity decreased significantly in the fourth postnatal week compared to the preceding Postnatal Week 3 in late preterm infants.

### Individuality and mode of delivery significantly explain variation in mycobiota composition

To investigate which clinical variables explained variation in the fecal mycobiota composition of preterm and full-term infants, PERMANOVA and redundancy analysis were performed. The differences between centroids of gestational age groups were assessed by PERMANOVA and differences were statistically significant (*P* = 0.005, Supplementary Table 3). Therefore, gestational age groups were used to categorize infants in hierarchical clustering and redundancy analysis. Hierarchical clustering was performed to investigate relatedness of samples in their respective gestational age categories. Samples of all gestational age categories did not cluster based on unweighted UniFrac distance of the mycobiota [Supplementary Figure 8]. Results of hierarchical clustering were rather random and could indicate that individual variability is high as well as the need for a larger number of samples per gestational age category.

**Figure 4:**
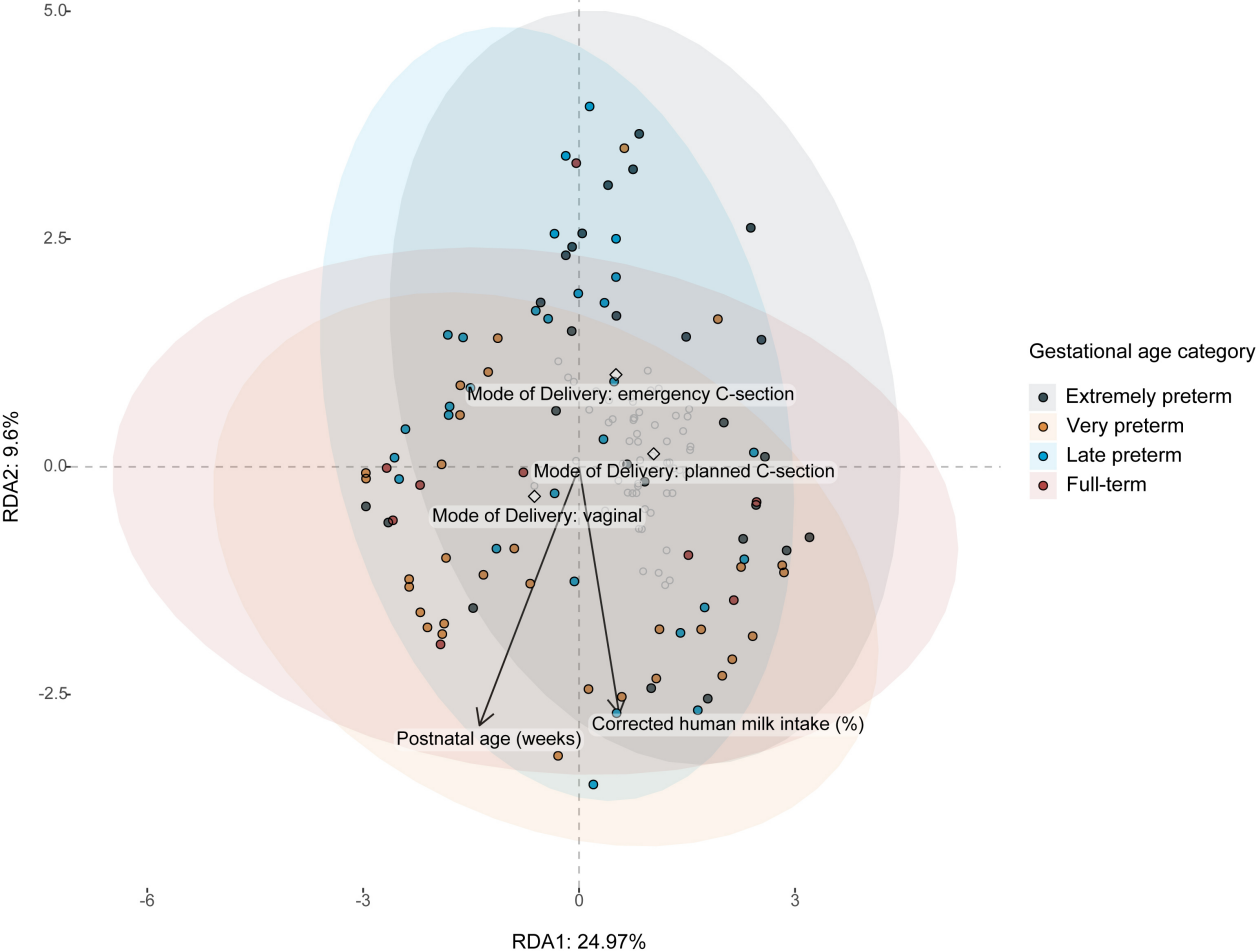
Redundancy analysis on the fecal mycobiota of preterm and full-term infants during the first six postnatal weeks. Continuous clinical variables are indicated with arrows, whereas the centroids of categorical clinical variables are indicated with diamonds. Mode of delivery was significant after automatic stepwise model selection (*P* = 0.005) and its centroids are displayed; centroids of other clinical variables were left out for clarity. Colored points indicate individual fecal samples colored within its respective gestational age category. The effect of gestational age categories on the mycobiota composition was verified with PERMANOVA analysis (*P* = 0.005, *P* = 0.005, Supplementary Table 3).

Subsequently, redundancy analysis was performed to investigate the effect of clinical variables on the variation of the mycobiota composition. The mode of delivery, gestational age, birth weight, individuality, duration of the second and third antibiotic treatment, and body weight contributed to explaining the variation in mycobiota composition before automatic stepwise model selection. After automatic stepwise model selection, individuality and mode of delivery were predictors significantly explaining variation in the mycobiota composition (*P*_individuality_ = 0.005, *P*_MoD_ = 0.005) *P* = 0.005, [Supplementary Table 4]. However, these predictors lost their significance after adjusting the *P*-value (*P*_adj.individuality _= 0.238 and *P*_adj.MoD_ = 0.238). Subsequently, the effect of individuality was removed to further investigate the effect of other clinical variables [Figure 4]. Here, mode of delivery did not significantly explain variation in mycobiota composition.

### Vaginal and caesarean delivery enrich for vaginal-like and skin-like fungi in preterm infants

**Figure 5:**
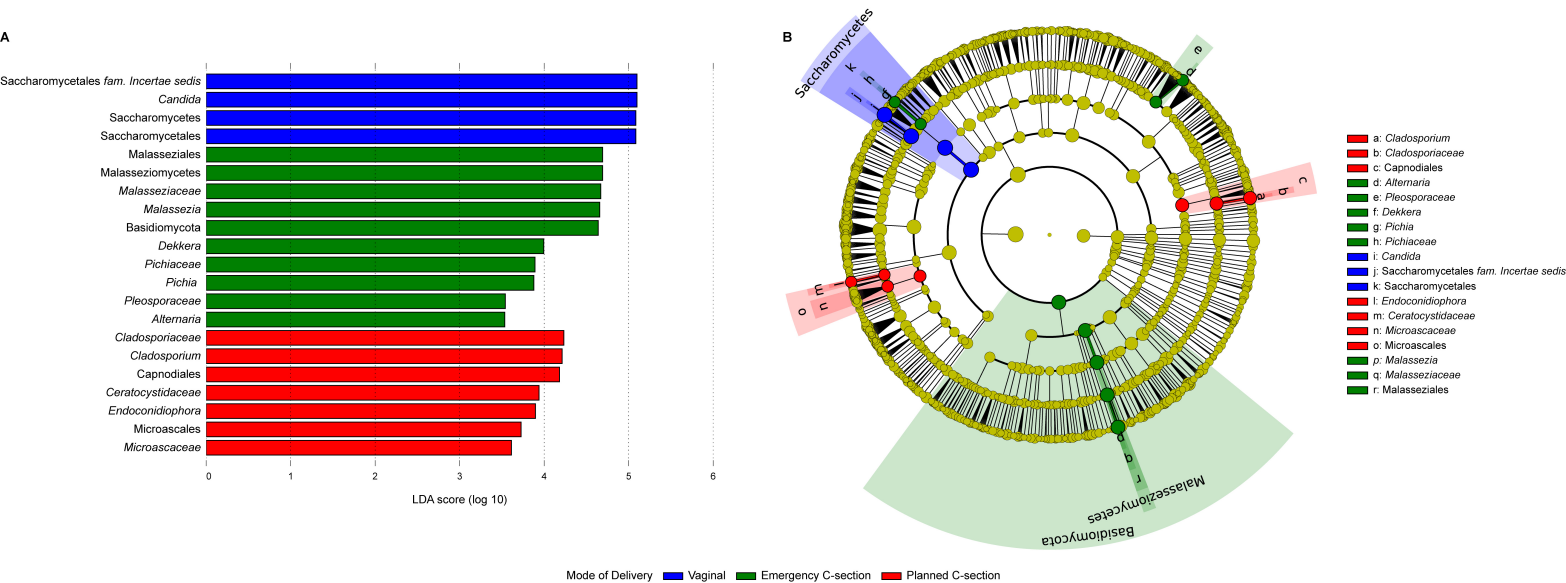
Linear discriminant analysis Effect Size results on the mode of delivery in preterm infant feces (*n* = 96). The fungal taxa that were significantly different in abundance between the mode of delivery groups are displayed in (A) a histogram of the LDA scores and (B) a cladogram.

Being significant initially in the redundancy analysis, the mode of delivery was hypothesized to influence mycobiota seeding. Vaginal delivery in particular is known to vertically transfer *Candida* spp. As such, we investigated the effect of mode of delivery on the mycobiota composition solely in preterm infants with LEfSe [Figure 5A and B]. Each type of delivery mode was characterized by specific taxa, with no overlap in taxa characteristic for planned and emergency caesarean (C-)sections [Figure 5B]. Instead, vaginally delivered infants indeed were enriched with the *Candida* genus. On the other hand, the Malasseziomycetes class and lower taxonomic levels were mainly characteristic for infants delivered through emergency C-section. Interestingly, the vaginally delivered and emergency C-section infants shared fungi within the Saccharomycetes class but not for lower taxonomic levels. Infants delivered with a planned C-section were, among others, enriched in the Microascales order and *Cladosporium* genus.

## DISCUSSION

Our findings show that preterm and full-term infants are colonized by various eukaryotic kingdoms, of which fungi were most prominent. Fungal diversity remained stable in the first six postnatal weeks and the genus *Candida* was the most abundant. Its abundance was additionally shown to increase with gestational and postnatal age. Although gestational age was important for the mycobiota composition, samples did not cluster based on gestational age categories. Instead, individuality and mode of delivery were significant predictors for mycobiota variation. Vaginally delivered infants were characterized by high abundance of *Candida* spp., whereas infants delivered through emergency C-section were characterized by *Malassezia* spp. Although the mycobiome is gaining more attention recently, this is the first time that the effect of clinical variables on the gut mycobiota composition is described for preterm infants with varying degrees of prematurity. We speculate these findings are relevant for clinical practice and will gain traction in the near future.

Interestingly, many other eukaryotic kingdoms were observed besides fungi. After fungi, the next most abundant kingdom was Viridiplantae. This kingdom has been observed more often in infants and has been suggested to be remnants from plant material ingested by the mother^[14,43]^. In fact, green algae are part of this kingdom and are used to generate supplements such as docosahexaenoic acid (DHA, omega-3)^[44]^. Omega-3 is essential for fetal neurodevelopment and is recommended in pregnancy and during breastfeeding^[44,45]^. We therefore hypothesize that parts of this eukaryotic DNA may end up in human milk and is thus transferred to the infant. Moreover, mother’s own milk was increasingly supplemented with human milk fortifier (Nenatal BMF) as part of standard neonatal care practices in Dutch NICUs, starting at 100 mL/kg/day enteral feeding. Fortification of human milk is necessary to meet the nutritional needs of the preterm infant. Human milk fortifier contains - besides protein, minerals, and vitamins - DHA, which might well be the origin of the detected Viridiplantae.

Within the fungal kingdom, the phyla Ascomycota and Basidiomycota were the most abundant in the infant intestine, which has also been observed previously^[46]^. Moreover, the abundance of *Candida* spp. increased with gestational and postnatal age. Similar to findings of James *et al*.^[11]^, we observed the *Candida* genus and the species *C. albicans* were most dominant in preterm infants. Interestingly, the abundance of the *Candida *genus was reported in lower abundance by James *et al.*^[11]^ Most preterm infants of that study did not receive antibiotic treatment after the second day of life, which suggests antibiotic treatment may have enriched *Candida* spp. in the preterm infants described herein. However, other confounding factors including the sampling period, mode of delivery, gestational age, and postnatal age should be accounted for in future studies to assess the effect of antibiotic treatment on mycobiota development.

While previous research highlighted the abundance of *Candida* spp.^[11]^, we were additionally able to show that vaginal delivery promotes colonization with *Candida* spp. Vertical transfer of this genus has been described and is therefore very likely to occur in infants described herein^[6,7]^. Although *Candida* spp. is commensal in most cases, the genus may also cause disease in immunocompromised hosts. Preterm infants often experience overgrowth of an opportunistic pathogenic fungus after antibiotic treatment, typically invasive systemic candidiasis^[19,47-49]^. Invasive systemic candidiasis in preterm infants can lead to considerable morbidity and mortality rates^[50-52]^. It is most often caused by *C. albicans*, which interestingly was the most abundant *Candida* species during all postnatal weeks. It might transition from commensal to opportunistic pathogen in response to perturbations in the microbiota and weakening of the immune system or of the physiologic barriers^[49,53-56]^. Factors that might trigger the transition include long-term or repeated use of broad-spectrum antibiotics, use of central venous catheters, parenteral nutrition, and a naïve immune system^[48,50,53]^. Indeed, antibiotics may promote overgrowth by *Candida* spp. through induction of genetic changes leading to increased fitness of *C. albicans *in the gut^[57]^. All infants in our cohort received at least one antibiotic treatment, were predominantly colonized by *Candida* spp. and the most abundant species was *C. albicans*. However, candidiasis was not observed in the current cohort. Hence, the mycobiome may act as reservoir for opportunistic pathogens in immunocompromised hosts such as preterm infants, which may be triggered by specific environmental influences such as antibiotic treatment^[58]^.

Individuality and mode of delivery were observed as significant predictors for mycobiota variation. Similar to our results, infant mycobiomes from anal swabs exhibited high intra-individual variation and thereby were concluded to be individualized^[8]^. Moreover, mode of delivery has previously been shown to shape the mycobiome composition in human milk as well as on skin, oral, and anal body sites of infants^[8,59]^. As hypothesized before^[9]^, we observed that vaginally delivered infants were enriched in *Candida* spp., whereas infants delivered through (emergency) C-section were characterized by *Malassezia* spp. *Malassezia* spp. are commonly identified on the skin of adults and infants and therefore have been hypothesized to be vertically transmitted from parent to infant upon skin contact^[60,61]^. In C-section infants, the gut microbiota composition has already been described to be more similar to mother’s skin microbiota^[62]^. Our data support the hypothesis of vertical transmission of fungi and thereby underpin the importance of the mode of delivery in bacterial and fungal colonization. However, *Malassezia* spp. was not characteristic for infants born through planned C-section. It remains unknown what has contributed to these differences as most studies lack distinction between types of caesarean delivery.

The question remains if the observed fungi are residents of the gut or rather transients. Fungi are present in relatively low concentrations of 10^5^-10^6^ cells per gram of fecal matter, although these numbers may be an underestimation^[15,63,64]^. Even though they are smaller in cell counts, fungal cells are 10-fold longer and 100-fold larger in volume than bacterial cells. Hence, the fungal biomass and the metabolites they produce cannot be compared with the microbiota by solely considering cell counts^[46]^. It is plausible that fungi are able to perform bioactive functions in the preterm gut, as metabolic, trophic, and protective functions have been described^[1]^. The same cohort of preterm and full-term infants has been studied previously by metaproteomics^[20,21]^. Here, we did detect *Candida*-derived proteins sporadically in gastric aspirates and feces. With advances in technology, we may now identify more proteins to better approximate fungal activity in the intestinal tract of infants. Therefore, future studies should elucidate activity of the mycobiota by investigating fecal proteomes of infants with state-of-the-art techniques that enable to identify more proteins.

While our study identified prominent fungi in the intestine of preterm infants over time and assessed which clinical variables influence the mycobiota composition, we acknowledge the relatively small number of particularly full-term infants and the lack of longitudinal data for some of the infants described in this study. This should be considered when interpreting the data and the significant outcomes, particularly when studying the differences in mycobiota composition per gestational age category. Additionally, the fungal load was not assessed by means of quantitative PCR due to insufficient sample material, which is needed to put the results into perspective of the intestinal ecosystem. Furthermore, sequencing the mycobiota has its challenges. Such challenges include the lack of a standardized and reliable method of mycobiota sequencing, as well as a more comprehensive fungal database coverage compared to bacterial databases^[65-67]^. Therefore, some taxa may have been over- or under-represented in the results described herein. Future studies should focus on developing standardized and reliable methods to allow scalability^[65]^. Subsequently, this may advance research of interkingdom interactions that are currently limited. These interactions are expected to be of great importance in a key body site where cross-talk and interactions with host immunity result in systemic manifestations of either health or disease^[65]^.

In conclusion, our findings indicate that fungi and other eukaryotic kingdoms can be detected in the intestine of preterm and full-term infants in the first six postnatal weeks. While intestinal fungi have been characterized in preterm infants before, this is the first time it was assessed in relation to clinical variables in preterm infants. The mycobiota shows great similarities in how individuality, mode of delivery, and gestational and postnatal age drive its development in preterm infants. As mycobiome research is gaining traction, future studies should focus on bridging the gap between the bacterial and fungal kingdoms in the gut. Such insights could refine the healthcare of this vulnerable group of infants.

## DECLARATIONS

### Availability of data and materials

Raw sequences with barcode and primer removed and supporting metadata were deposited in the European Nucleotide Archive (http://www.ebi.ac.uk/ena) under the accession number PRJEB48004.

### Copyright

© The Author(s) 2022.
