# Supplementary material for: The first fungi: mode of delivery determines early life fungal colonization in the intestine of preterm infants
Source: Microbiome Res Rep. 2022 Jan 28;1(1):7. doi: 10.20517/mrr.2021.03 (PMC10714301; doi:10.20517/mrr.2021.03)
Supplement: Supplementary file 1 [file mrr-1-1-7-SupplementaryMaterials.pdf]

Supplementary Material:

The first fungi: mode of delivery determines early life fungal colonization in the intestine of preterm infants

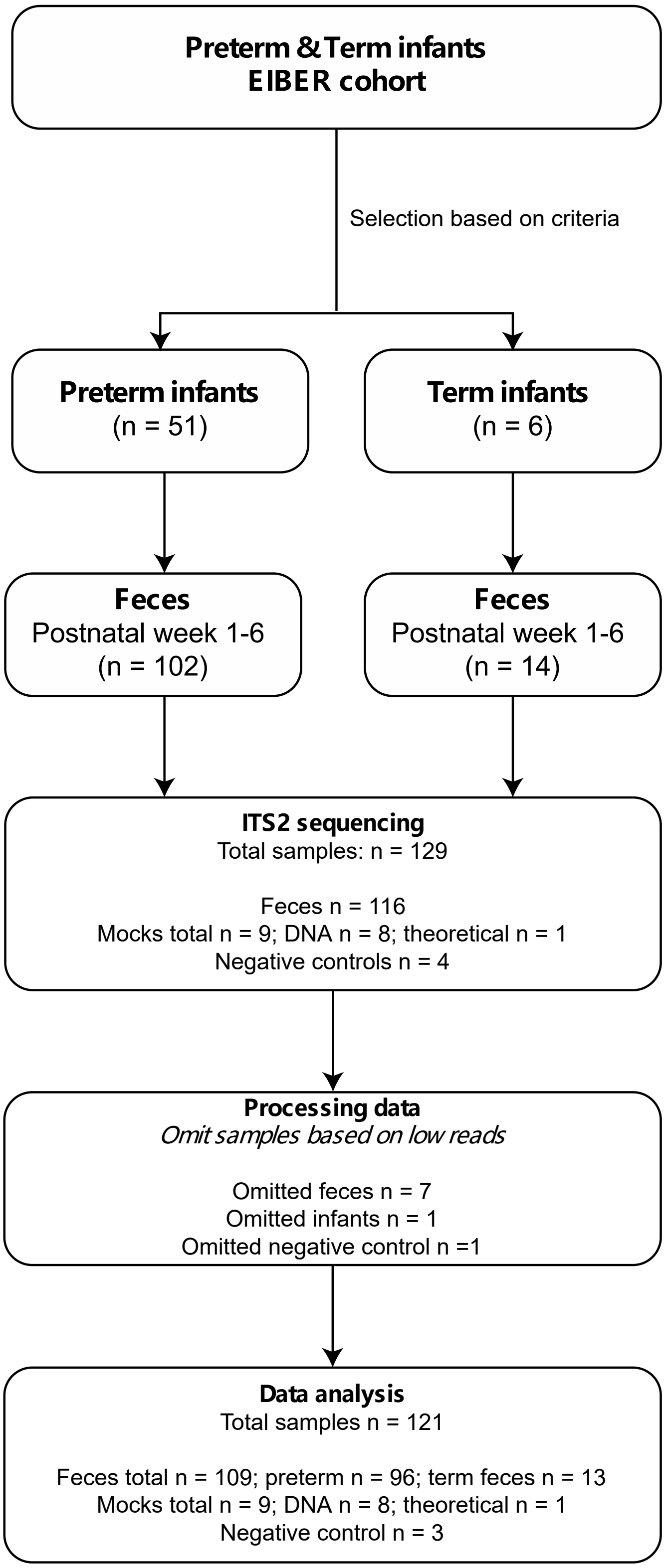

Supplementary Figure 1. Overview and workflow diagram of this study. Preterm and

full-term infants were part of the EIBER study, in which feces were collected in the first six postnatal weeks with exception of Week 5. Samples of the current study were selected based on the sample selection criteria and were used for ITS2 sequencing.

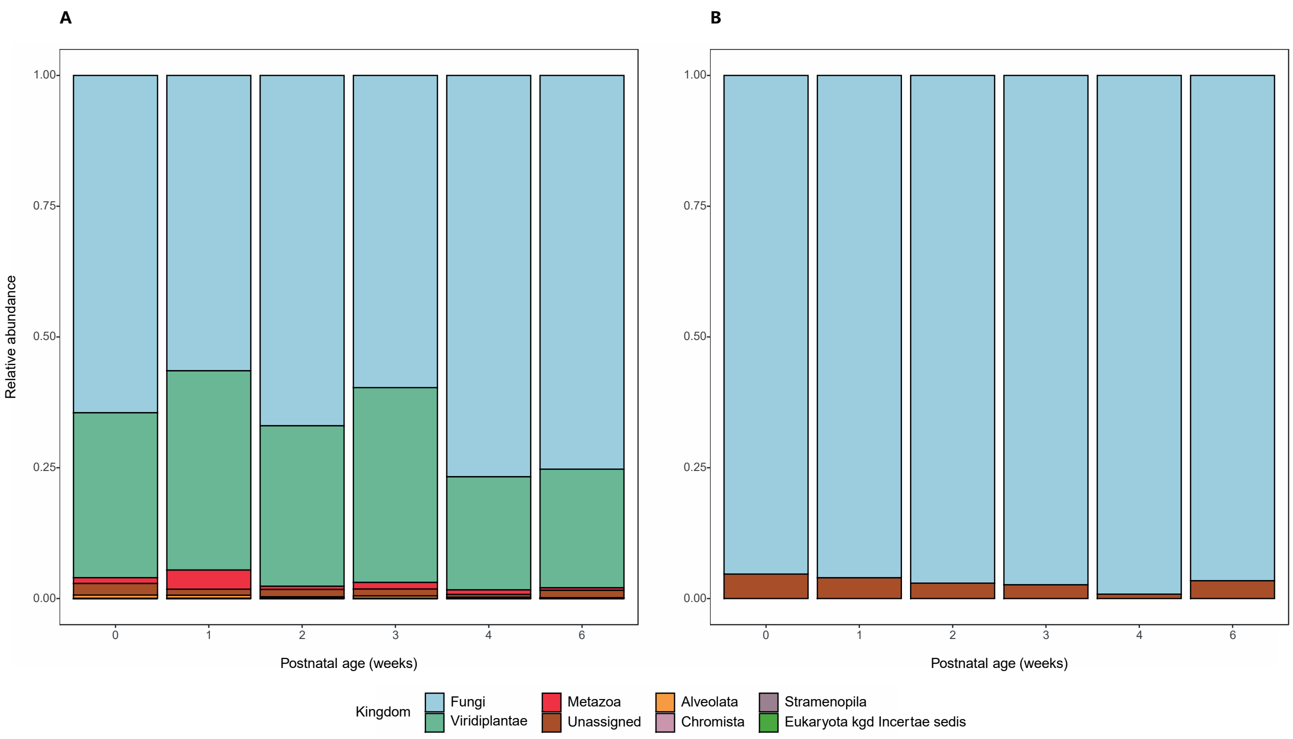

Supplementary Figure 2. Relative abundance of all kingdoms identified in feces of preterm and full-term infants in the first six postnatal weeks. The data is displayed (A) before and (B) after pre-processing.

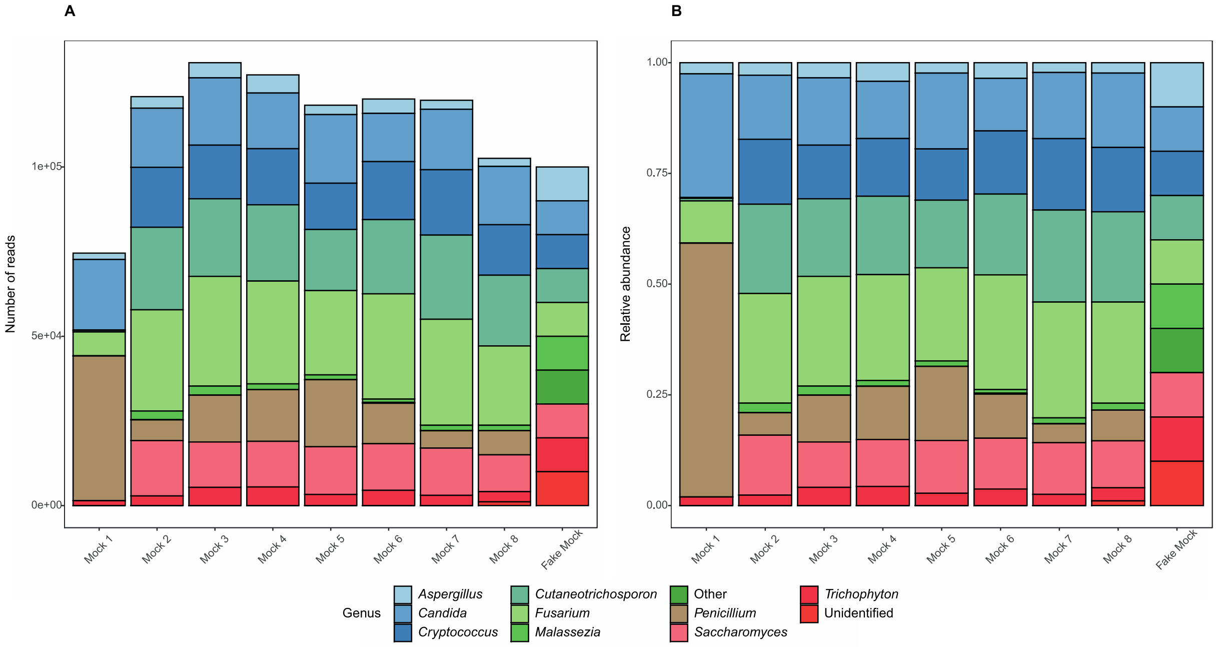

Supplementary Figure 3. DNA and fake mock community composition plots of the ten most abundant genera. The DNA and fake mock communities in (A) number of reads and (B) relative abundance.

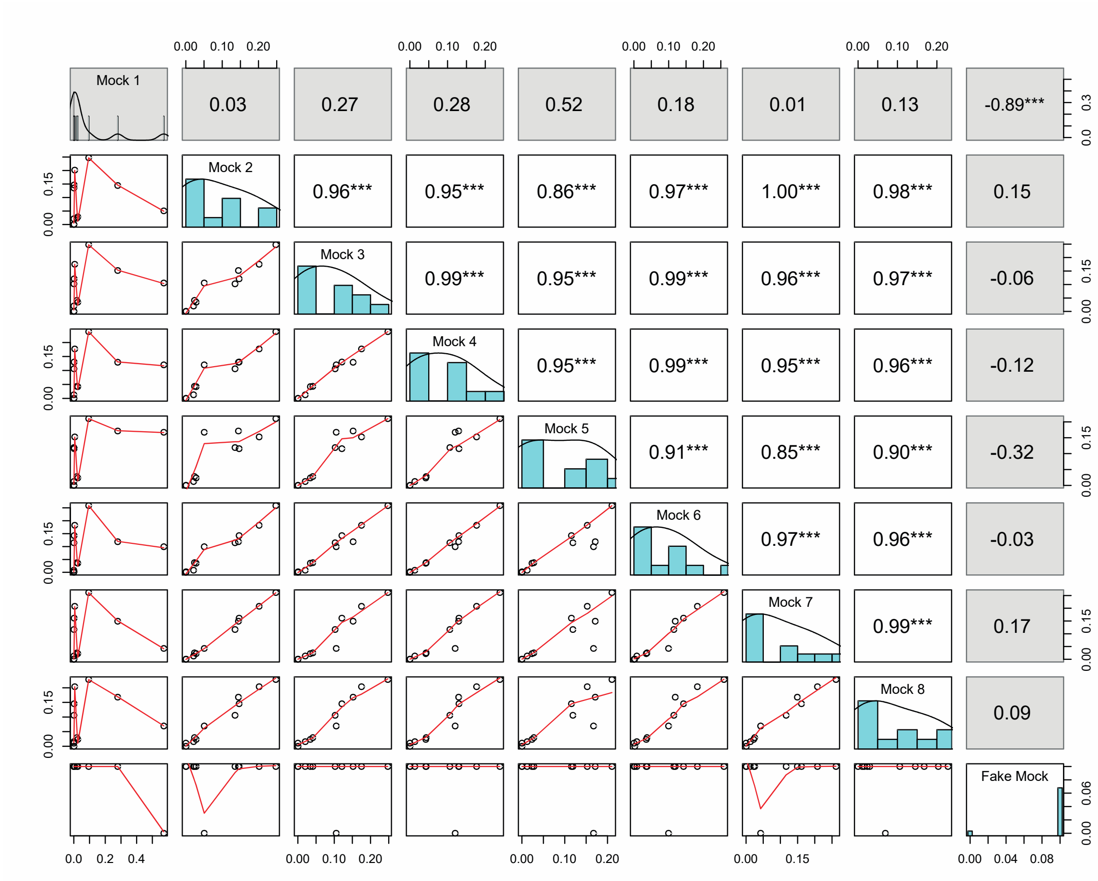

Supplementary Figure 4. Correlation matrix of DNA and fake mock communities. The correlation coefficients of Mock 2-8 were used for quality control, whereas correlation

coefficients of Mock 1 were not considered due to deviant absolute reads (indicated in grey). Asterisks indicate significance levels with $***P \leq 0.001$.

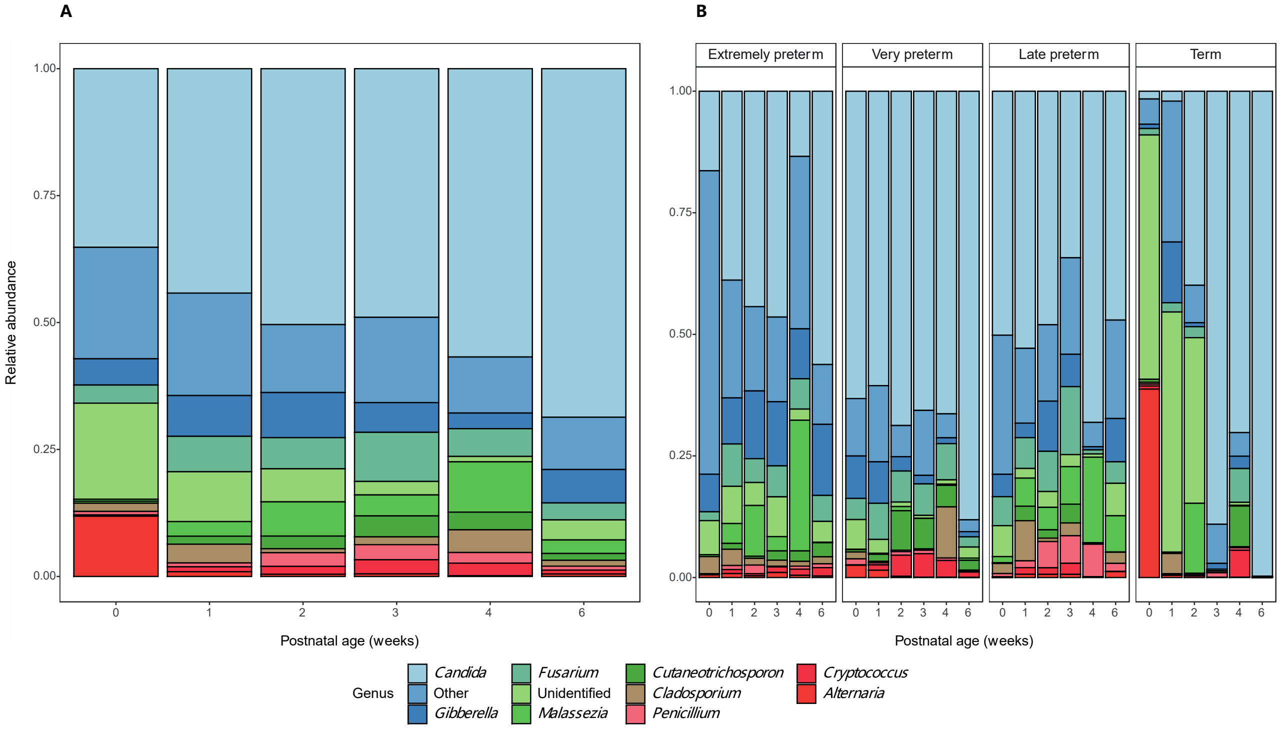

Supplementary Figure 5. Relative abundance of the ten most abundant genera in feces of preterm and full-term in the first six postnatal weeks. Feces of (A) all preterm and full-term infants together; and (B) preterm and full-term infants in their gestational age categories.

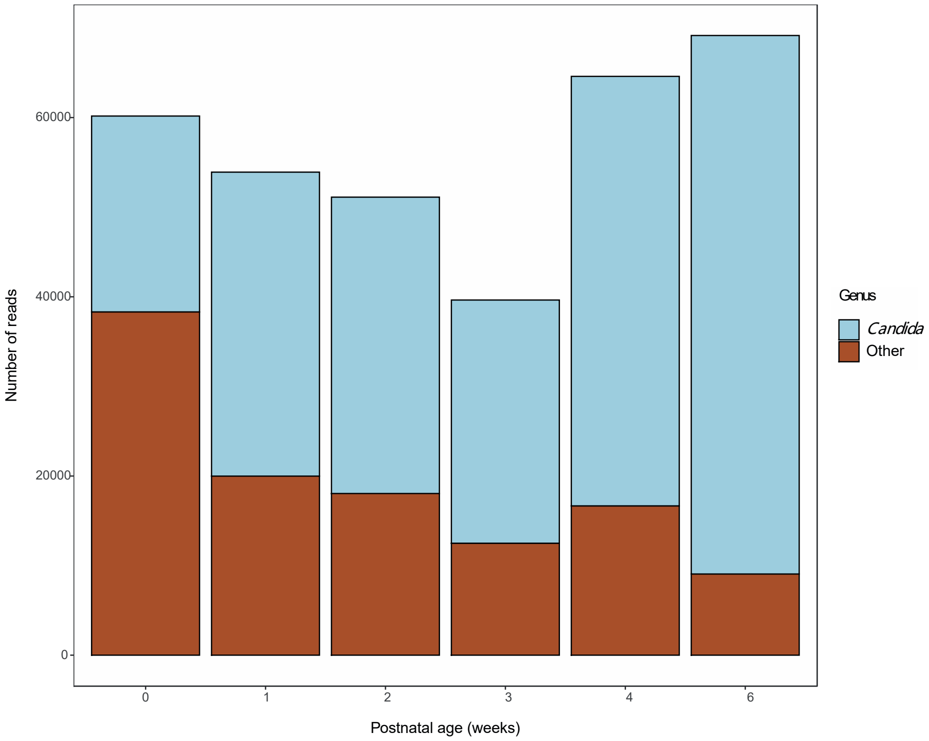

Supplementary Figure 6. Number of reads of *Candida* spp. relative to the other genera identified in feces of all preterm and full-term infants together in the first six postnatal weeks.

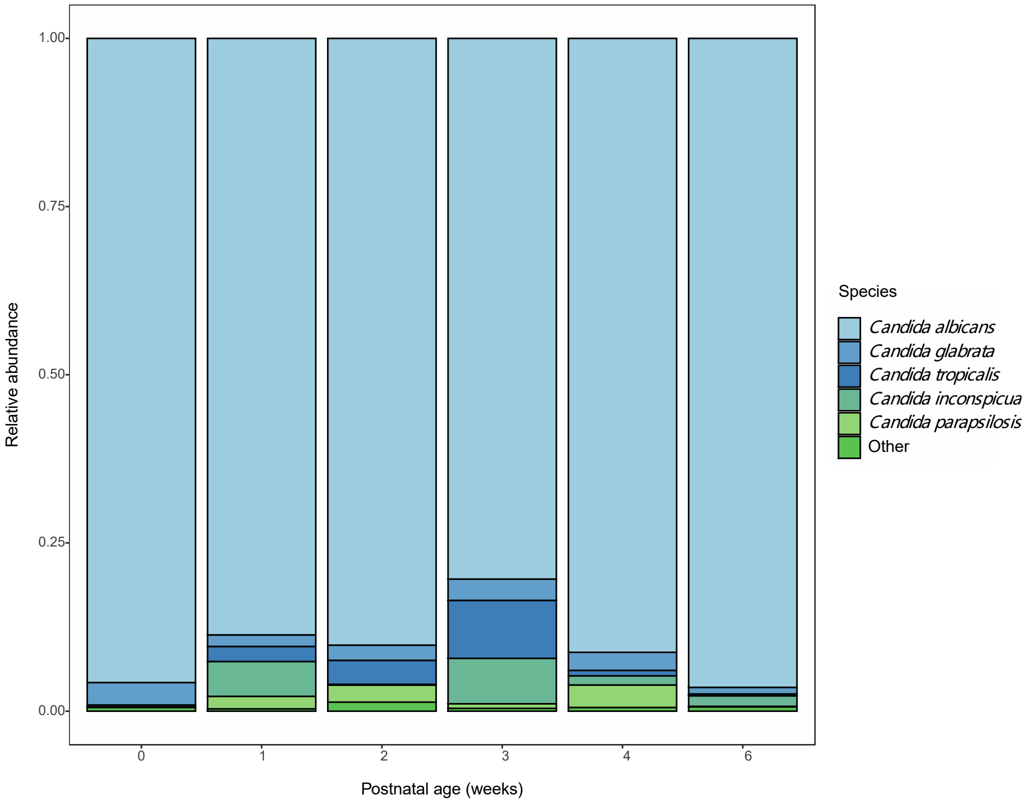

Supplementary Figure 7. Relative abundance of the five most abundant species within the *Candida* genus for all preterm and full-term infants in the first six postnatal weeks.

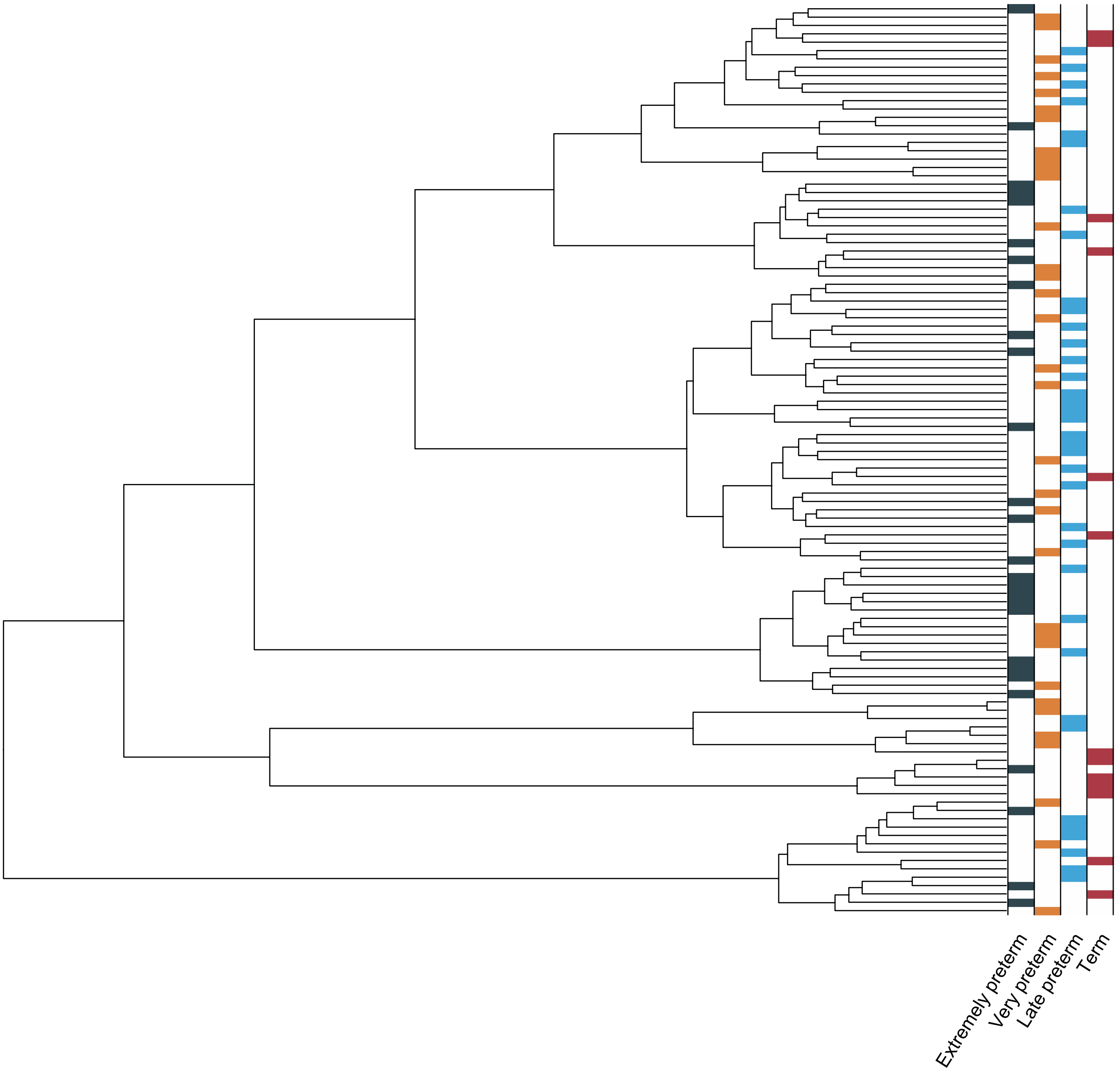

Supplementary Figure 8. Hierarchical cluster dendrogram of feces of preterm and full-term infants in their gestational age categories. The distance is based on unweighted UniFrac, and clustering is performed with Ward. The bars indicate a sample belongs to the gestational age category when colored.

Supplementary Table 1. Characteristics of the infants used for data analysis

	**Total infants**	**Total samples[a]**	**Gestational age (mean ± SD)**	**Birth weight (grams)**	**Female (%)**	**C-section (%)**	**Enteral feeding (%)[a]**	**Human milk (%)[a,b]**	**≥ 2 antibiotic treatments (%)**	**Antimycotics (%)**
All	56	109 (7, 17, 28, 19, 20, 18)								
EP	18	27 (1, 6, 9, 3, 4, 4)	26.3 ± 0.9	855.2 ± 207.7	44.4%	66.7%	31; 76; 76; 95; 100	25; 65; 76; 95; 100	77.8%	61.1%
VP	15	33 (2, 5, 6, 6, 8, 6)	29.2 ± 1.1	1387.3 ± 260.1	46.7%	46.7%	54; 91; 100; 88; 100	47; 75; 83; 85; 50	40.0%	26.7%
LP	17	36 (2, 4, 10, 9, 5, 6)	33.8 ± 1.1	2254.6 ± 506.0	35.3 %	23.5 %	78; 98; 100; 100; 100	54; 48; 45; 36; 25	11.8%	41.2%
FT	6	13 (2, 2, 3, 1, 3, 2)	38.0 ± 1.3	3279.6 ± 555.0	16.7%	33.3%	100; 100; 100; 100; 100	38; 78; 90; 91; 44	0.0%	33.3%

Characteristics of the selected infants from the EIBER cohort of whom feces were used to perform data analysis: 56 infants in total of whom 50 are preterm and six are term. Feces of the first six postnatal weeks were collected with exception of postnatal week five. [a]The percentage at postnatal weeks 1, 2, 3, 4 and 6 respectively. [b]The percentage of human milk was corrected for the amount of enteral feeding. EP: Extremely preterm; VP: very preterm; LP: late preterm; FT: full-term.

Supplementary Table 2. Scheme of samples available per infant for characterization of the inestinal fungal community

**Infant ID**	**Gestational age**	**Gestational age group**	**Week 0**	**Week 1**	**Week 2**	**Week 3**	**Week 4**	**Week 6**	**Number of samples**
**A001**	**27**	**EP**	0	1	1	0	0	0	2
**A003**	**25**	**EP**	0	1	1	0	0	0	2
**A007**	**28**	**VP**	0	0	0	0	0	1	1
**A008**	**28**	**VP**	0	0	1	1	0	0	2
**A012**	**27**	**EP**	0	0	1	0	0	0	1
**A015**	**31**	**VP**	1	1	0	0	1	1	4
**A019**	**26**	**EP**	0	0	0	0	0	1	1
**A020**	**28**	**VP**	1	0	1	0	1	1	4
**A021**	**28**	**VP**	0	0	0	1	1	1	3
**A022**	**27**	**EP**	0	0	0	0	0	1	1
**A028**	**30**	**VP**	0	1	0	0	0	0	1
**A030**	**26**	**EP**	0	0	1	0	0	0	1
**A031**	**26**	**EP**	0	1	0	0	0	0	1
**A032**	**24**	**EP**	0	1	0	0	0	0	1
**A037**	**29**	**VP**	0	0	1	0	1	0	2

**A038**	**30**	**VP**	0	1	1	0	1	0	3
**A041**	**26**	**EP**	0	1	0	0	0	0	1
**A043**	**27**	**EP**	0	0	1	0	1	1	3
**A044**	**28**	**VP**	0	1	0	0	1	0	2
**A047**	**27**	**EP**	0	0	1	0	0	0	1
**A050**	**30**	**VP**	0	0	1	1	0	0	2
**A051**	**30**	**VP**	0	0	1	1	1	0	3
**A056**	**30**	**VP**	0	1	0	0	0	0	1
**A063**	**25**	**EP**	0	0	1	1	0	0	2
**A068**	**27**	**EP**	0	0	0	1	1	0	2
**A074**	**26**	**EP**	0	0	1	0	0	0	1
**A076**	**27**	**EP**	0	1	0	0	0	1	2
**A082**	**25**	**EP**	1	0	1	0	0	0	2
**A095**	**27**	**EP**	0	0	0	0	1	0	1
**A097**	**27**	**EP**	0	0	0	1	1	0	2
**A102**	**29**	**VP**	0	0	0	0	0	1	1
**A103**	**29**	**VP**	0	0	0	1	1	1	3

Henderickx *et al. Microbiome Res Rep* Year;Volume:Number | http://dx.doi.org/10.20517/mrr.xxxx.xx

**A108**	**28**	**VP**	0	0	0	1	0	0	1
**B202**	**33**	**LP**	0	0	1	0	0	1	2
**B211**	**32**	**LP**	0	0	1	1	0	1	3
**B212**	**32**	**LP**	0	0	1	0	0	0	1
**B214**	**40**	**FT**	0	0	0	0	1	0	1
**B216**	**37**	**FT**	0	1	0	1	1	1	4
**B217**	**34**	**LP**	1	0	0	0	0	0	1
**B233**	**39**	**FT**	1	0	0	0	1	0	2
**B234**	**36**	**LP**	0	0	1	0	1	1	3
**B237**	**33**	**LP**	0	0	0	1	1	0	2
**B245**	**34**	**LP**	0	1	1	1	0	1	4
**B246**	**34**	**LP**	0	0	1	0	0	0	1
**B248**	**34**	**LP**	0	0	0	1	1	1	3
**B254**	**32**	**LP**	0	0	1	1	0	0	2
**B266**	**35**	**LP**	0	0	1	1	1	1	4
**B270**	**39**	**FT**	0	0	1	0	0	1	2
**B280**	**37**	**FT**	1	1	1	0	0	0	3

**B285**	**34**	**LP**	1	1	1	1	0	0	4
**B300**	**34**	**LP**	0	1	0	1	0	0	2
**B308**	**33**	**LP**	0	0	1	0	0	0	1
**B310**	**40**	**FT**	0	0	1	0	0	0	1
**B313**	**35**	**LP**	0	1	0	0	0	0	1
**B316**	**33**	**LP**	0	0	0	1	0	0	1
**B318**	**34**	**LP**	0	0	0	0	1	0	1
								**Total:**	**109**

Available samples are indicated with “1”, while unavailable/insufficient samples are indicated with “0”. EP: Extremely preterm; VP: very preterm; LP: late preterm; FT: full-term.

Supplementary Table 3. PERMANOVA analysis.

**A**	**Df**	**Sum of Squares**	**Mean Squares**	**F Model**	**R2**	**Pr(>F)**
Gestational age category	3	3202	1067.4	2.05	0.06	0.005**
Residuals	91	47,502	522.0		0.94	
Total	94	50,705			1.00	

Henderickx *et al. Microbiome Res Rep* Year;Volume:Number | http://dx.doi.org/10.20517/mrr.xxxx.xx

**B**	**Df**	**Sum Squares**	**Mean Squares**	**F value**	**Pr(>F)**
Groups	3	57	19.06	0.85	0.47
Residuals	91	2,029	22.29		

(A) The results of PERMANOVA analysis based on gestational age categories; and (B) the results of the associated check for assumed homogeneity of variances. **$P \leq 0.01$.

Supplementary Table 4. Tables of RDA data

**A**	**Df**	**AIC**	**F**	**Pr(>F)**	**P_{adj}**
Mode of Delivery	2	594.9	3.902	0.005**	0.238
Individuality	53	597.9	1.440	0.005**	0.238

**B**	**RDA1**	**RDA2**
Eigenvalue	134.6732	51.7781
Proportion explained	0.2497	0.0960
Cumulative proportion	0.2497	0.3457

(A) The ANOVA table and (B) accumulated constrained eigenvalues. Scaling 2 for species and site scores. Species are scaled proportional to eigenvalues. Sites are unscaled: weighted dispersion equal on all dimensions. General scaling constant of scores: 15.0059. **$P \leq 0.01$, with P-values adjusted with Benjamini-Hochberg.
